# The mediation role of hope in the relationship of resilience with depression, anxiety, and stress in caregivers of children and adolescents with cancer

**DOI:** 10.1038/s41598-024-65922-4

**Published:** 2024-07-09

**Authors:** Masoume Rambod, Fatemeh Nassabeh, Mahdi Salmanpour, Nilofar Pasyar

**Affiliations:** 1 Community Based Psychiatric Care Research Center, Nursing and Midwifery School, Shiraz University of Medical Sciences, Shiraz, Iran; 2 Department of Psychology, Shiraz University, Shiraz, Iran; 3 Department of Statistics, College of Mathematical Sciences, University of Kashan, Kashan, Iran

**Keywords:** Anxiety, Cancer, Caregiver, Depression, Hope, Resilience, Cancer, Psychology, Oncology

## Abstract

This study aimed to determine the mediation role of hope in the relationship of resilience with depression, anxiety, and stress in caregivers of children and adolescents with cancer. This cross-sectional study was conducted on 200 caregivers of children and adolescents with cancer. Adult Hope Scale, Connor-Davidson Resilience Scale, and Depression, Anxiety and Stress (DAS) scales were used for data collection. The mediator and moderator model was tested using the SPSS macro PROCESS (Model 4, and 5). The mediator model (model 4) indicated that DAS significantly correlated with resilience (β = − 0.54, t-value = − 5.01, *p* < 0.001), and hope (β = − 0.84, t-value = − 3.45, *p* = 0.0007). Hope mediated the relationship between resilience and DAS among caregivers of children and adolescents with cancer (Effect = − 0.18, SE = 0.06, 95% CI − 0.33 to − 0.06). The mediator and moderator model (model 5) showed that female caregivers had a stronger correlation between resilience and DAS when compared to their male counterparts (β = − 0.56, t-value = − 3.90, *p*-value = 0.0001); also, hope mediated the relationship between resilience and DAS among caregivers of children and adolescents with cancer (Effect = − 0.20, SE = 0.08, 95% CI − 0.37 to − 0.04). In conclusion, hope was a mediator, and female caregivers were a moderator in the relationship of resilience with depression, anxiety, and stress, and its promotion might be effective among caregivers of children and adolescents with cancer. It seems that resilience, female caregivers, and hope may provide good protection against depression, anxiety, and stress in caregivers of cancer patients.

## Introduction

Childhood cancer affects family functioning^1^, relationships, and quality of life^2^. It was reported that caregivers of children with cancer had a moderate to high burden of care^3^. Moreover, these caregivers had cognitive, social, and physical problems^4^. Children’s symptom burden predicted the parental stress and led to lower caregivers’ quality of life^5,6^. Caregivers of children with cancer experienced high levels of stress, which led to worse psychological adjustment ^7^. Besides the stress, approximately half of the pediatric cancer patients’ caregivers suffered from depression^8^. In addition, the majority of them reported distress^4^, anxiety, and depression disorders^9^.

Promoting resilience was an effective stress management in caregivers of children with cancer^10^. Resilience is the capacity to react to stress in a healthy condition where goals are achieved with negligible negative psychological and physical effects ^11^. It plays an important role in adapting cancer patients’ caregivers to stressful situations^12^. Paying attention to resilience is a critical component of cancer care^13^ because resilience improves cancer patients’ quality of life^14^.

Moreover, caregivers of cancer patients were hopeful to treat and cure the illness and prolong the survival span. Snyder et al.^15^ theory defines hope as “a dynamic motivational experience that is interactively derived from two distinct types of cognitive tools in the context of goal achievement–namely pathways and agency thinking”. Based on Snyder’s hope theory, hopeful people have goal-oriented thoughts, develop strategies to achieve the goals, and are motivated to expend effort to achieve goals ^15^. Therefore, hope, as a dynamic motivational experience, may positively affect the family members’ activities^16^ and quality of life^17^. Hope also decreased the caregivers’ burden^17^. Hope is negatively correlated with depression^18^.

As mentioned above, caregivers of cancer patients may experience depression, anxiety, and stress, but resilience and hope, as a coping resource, may be effective in reducing depression, anxiety, and stress in these caregivers. The question is whether there is a relationship of resilience with depression, anxiety, and stress (DAS) in caregivers of children and adolescents with cancer. Few studies reported that resilience was associated with depression and anxiety in caregivers of children and adolescents with cancer^19,20^. Moreover, it was shown that higher resilience was associated with lower level of anxiety and depressive symptoms among family caregivers of patients with advanced cancer^21^.

**Figure 1:**
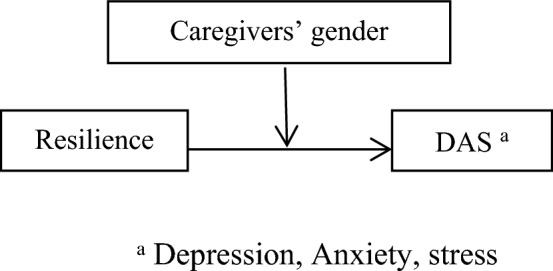
Hypothesised caregivers’ gender as moderate in the relationship between resilience, and DAS among caregivers of children and adolescents with cancer, based on the “Model 1”.

**Figure 2:**
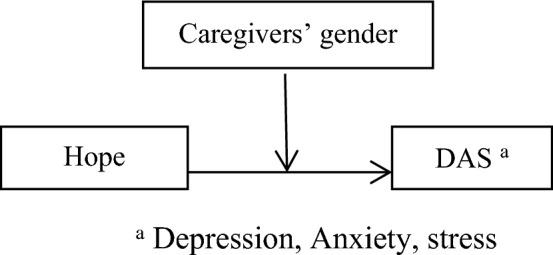
Hypothesised caregivers’ gender as moderate in the relationship between hope, and DAS among caregivers of children and adolescents with cancer, based on the “Model 1”.

**Figure 3:**
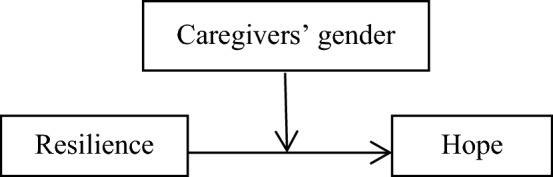
Hypothesised caregivers’ gender as moderate in the relationship between resilience,and hope among caregivers of children and adolescents with cancer, based on the “Model 1”.

**Figure 4:**
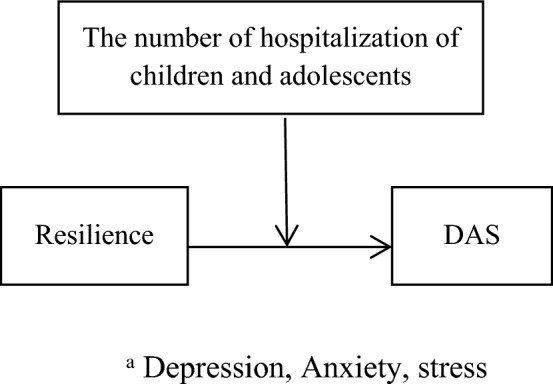
Hypothesised the number of hospitalization of children and adolescents as moderate in the relationship between resilience,and DAS among caregivers of children and adolescents with cancer, based on the “Model 1”.

**Figure 5:**
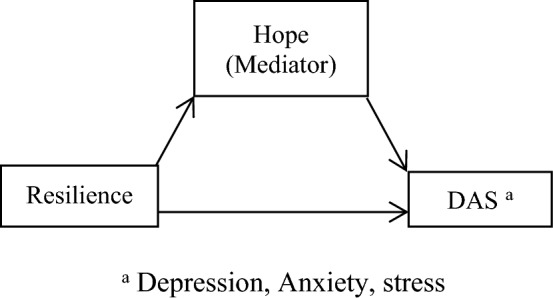
Hypothesised mediation role of hope in relationship of resilince and depression, anxiety, and stress among caregivers of children and adolescents with cancer, based on the “Model 4”.

**Figure 6:**
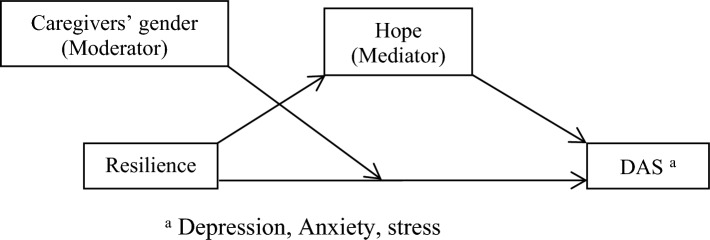
Hypothesised moderated mediation model of resilince and depression, anxiety, and stress among caregivers of children and adolescents with cancer, based on the “Model 5”.

Another question is whether hope plays a mediating role between resilience and DAS in caregivers of children and adolescents with cancer. A study showed that hope was a mediator in the relationship between resilience and positive coping in cancer patients^22^. Another study indicate that resilience is a dynamic and changeable path that improves hope and decreases depressive symptoms in cancer patients^23^. Another study showed that hope was a mediator in the association between resilience and body image distress in cancer patients^24^. The review of the literature showed that the answers to the above-mentioned questions were ambiguous; most of them focus on cancer patients, not on caregivers, and it is necessary to conduct more evidence-based studies on the relationship between resilience and DAS related to the caregivers of children and adolescents with cancer and the mediating role of hope. Moreover, we asked ourselves whether caregivers’ gender could moderate the relationship between resilience and DAS. One study reported that parents’ gender was not associated with their resilience, anxiety, and depression^25^. On the other hand, another study showed that the parents’ gender was a predictor of resilience^26^. To improve the evidence-based practice, we posed the following hypotheses:

1. Caregivers’ DAS will be associated with the demographic and clinical characteristics of the caregivers and their children and adolescents with cancer.
2. DAS (depression, anxiety, and stress) will correlate to resilience, hope, caregivers’ gender, and the number of hospitalizations of children and adolescents.
3. Caregivers’ gender will moderate the relationship between resilience, DAS, and hope (Figs. 1, 2, and 3).
4. The number of hospitalization of children and adolescents will moderate the relationship between the caregivers’ resilience, DAS (Fig. 4), and hope.
5. Hope will mediate the relationship between resilience and DAS among caregivers of children and adolescents with cancer (Fig. 5).
6. Caregivers’ gender will moderate the indirect effect of resilience on DAS through the mediating effect of hope (Fig. 6).

## Methods

### Design

This is a cross-sectional study.

### Setting and participants

Two hundred caregivers with children and adolescents with cancer were recruited from the outpatient chemotherapy center in Emam Reza clinic and hematology and oncology wards in Amir and Namazee hospitals affiliated with Shiraz University of Medical Sciences.

### Sampling

The caregivers participated in this study using convenience sampling.

### Inclusion and exclusion criteria

The inclusion criteria were the caregivers (father and mother) who acted as the parents who lived with the child and provided daily care to him/her; mothers/fathers with children aged 17 years old and younger; those with children being known cases of cancer; and passage of one month after cancer diagnosis. The caregivers who experienced a crisis such as divorce and the death of a close family member during the last six months were excluded from the study. We aimed to exclude these individuals from the study because the crises in their acute phase can negatively affect their psychological state. However, no one stated this; therefore, no one was excluded from the study. Moreover, we were supposed to exclude caregivers who were not biological parents; however, no one had this criterion.

### Sample size

In this study, a-priori sample size estimation was used. Based on the “Structural Equation Model Sample Size Calculator” website (https://www.analyticscalculators.com/calculator.aspx?id=89, 2022), β = 0.8, α = 0.05, number of latent variables = 6, number of observed variables = 58, and effect size (r^2^) = 0.274 of our pilot study from substantial level, the minimum sample size to detect the effect was estimated at 199 ≈ 200. It should be noted that a pilot study was conducted before the main study. It was done on 40 cancer caregivers.

### Measures

Demographic and clinical data were collected on the child’s gender and age, parents’ age, marital status and education, number of previous chemotherapies, and number of hospitalizations of children and adolescents.

Adult Hope Scale, which consisted of 12 items, was used^15^. Each item used an eight-point scale ranging from “definitely false” to “definitely true”^27^. This scale contained two subscales, namely agency (questions: 2, 9, 10, and 12) and pathway (questions: 1, 4, 6, and 8). Each subscale had four items (questions), ranging from 4 to 32. The total hope score ranged from 8 to 64. Higher scores showed higher hope levels. The scores of 40–48, 48–56, and > 56 indicate hopeful, moderate, and high hope, respectively. The concurrent and divergent validities of this scale were approved^28^.

Connor-Davidson Resilience Scale developed by Connor and Davidson in 1991–1997^29^ was used. It has 25 items scored through a five-point Likert scale ranging from zero to 100. Higher scores indicate more resilience^29^. Cronbach’s alpha coefficient of the Persian version of the scale was shown to be 0.96 in diabetic parents^30^.

Depression, Anxiety, and Stress Scale (DASS-21) was used. DASS consists of 21 items scored using a 4-point Likert scale. DASS-21 is a short form of DASS-42. Therefore, the final scores of each subscale and total scale need to be multiplied by two^31^. The structural validity of the scales was approved using confirmatory factor analysis^32^.

Data were collected in outpatient chemotherapy center in Emam Reza Clinic and hematology and oncology wards in Amir and Namazee hospitals.

### Data analysis

The data were analyzed using SPSS 24.0 and frequency, percentage, mean, and SD were reported. The hypothesized model of resilience among caregivers of children and adolescents with cancer was used to guide the analysis (Figs. 1, 2, 3, 4, 5, and 6). Based on this model, hope and caregivers’ gender (mother/father) were considered as a mediator and moderator, respectively. The models 1 and 4 of the SPSS macro PROCESS were used to test the moderation and mediation effect, respectively. The model 5 of SPSS macro PROCESS was used to assess the moderated mediation effect. A *p*-value < 0.05 was considered as significant.

### Ethics approval and consent to participate

This study was approved by the Ethics Committee of Shiraz University of Medical Sciences (IR.SUMS.REC.1395.14039). This study was in accordance with the Declaration of Helsinki. INFORMED consent was obtained from all the participants/legal guardians (caregivers of children and adolescents with cancer). The caregivers filled out the questionnaires anonymously, and they were assured of the confidentiality of the data and their voluntary participation in this study.

## Results

### Caregivers and children’s demographic and clinical characteristics

The mean age of the fathers, mothers, and their children with cancer was 39.98 (SD = 7.08), 35.05 (SD = 6.19), and 7.26 (SD = 4.5) years, respectively. As Table 1 shows, 71.5% of the caregivers were female. 55.5% of the mothers and 46.2% of the fathers had secondary and high school education. Moreover, 93.4% of the parents lived together.

**Table 1:** The demographic and clinical characteristics of caregivers and their children and adolescence with cancer and their association with caregivers’ DAS (depression, anxiety, and stress).

Variables	n (%)	DAS	Depression	Anxiety	Stress
		Mean (SD)	Mean (SD)	Mean (SD)	Mean (SD)
Caregiver’s gender					
Mother	123 (71.5)	45.60 (26.52)	13.72 (9.17)	13.54 (10.00)	18.31 (8.99)
Father	49 (28.5)	40.04 (26.68)	11.26 (9.16)	12.36 (9.58)	16.40 (9.91)
t, *p*		t^a^ = 1.23, *p* = 0.21	t^a^ = 1.58, *p* = 0.11	t^a^ = 0.70, *p* = 0.48	t^a^ = 1.21, *p* = 0.22
Caregiver’s marital status					
Parent lived together	183 (93.4)	44.16 (25.87)	13.33 (8.98)	13.25 (9.50)	17.51 (9.14)
Parent divorced	6 (3.1)	21.33 (5.16)	3.33 (2.06)	6.66 (1.03)	11.33 (2.06)
Wife or husband died	7 (3.6)	46.00 (32.74)	13.14 (11.71)	14.00 (10.95)	18.85 (11.48)
F, *p*		F^b^ = 2.30, *p* = 0.10	F^b^ = 3.60, *p* = 0.02*	F^b^ = 1.45, *p* = 0.23	F^b^ = 1.44, *p* = 0.23
Mother’s education					
Primary school	31 (15.7)	51.48 (28.40)	14.70 (9.07)	16.19 (11.57)	20.58 (9.55)
Secondary school	22 (11.1)	49.54 (28.65)	16.09 (10.48)	15.90 (8.80)	17.54 (11.15)
High school and diploma	88 (44.4)	42.44 (27.60)	11.97 (9.38)	13.25 (10.20)	17.23 (9.60)
Academic degree	57 (28.8)	39.57 (19.40)	12.70 (7.57)	10.21 (6.02)	16.66 (7.30)
F, *p*		F^b^ = 1.87, *p* = 0.13	F^b^ = 1.63, *p* = 0.18	F^b^ = 3.66, *p* = 0.01*	F^b^ = 1.33, *p* = 0.26
Father’s education					
Primary school	29 (15.8)	51.44 (24.18)	14.06 (7.45)	16.34 (9.10)	21.03 (9.77)
Secondary school	32 (17.4)	34.68 (23.10)	8.75 (8.06)	10.56 (8.69)	15.37 (8.26)
High school and diploma	53 (28.8)	41.25 (30.00)	13.20 (10.14)	13.24 (10.34)	14.78 (10.12)
Academic degree	70 (38.0)	40.74 (20.92)	12.48 (7.70)	11.08 (8.13)	17.17 (7.34)
F, *p*		F^b^ = 2.41, *p* = 0.06	F^b^ = 2.49, *p* = 0.06	F^b^ = 2.90, *p* = 0.03*	F^b^ = 3.48, *p* = 0.01*
Children and adolescent gender					
Girl	106 (55.2)	42.76 (24.17)	12.45 (8.56)	13.18 (8.99)	17.13 (8.43)
Boy	86 (44.8)	44.60 (27.56)	13.62 (9.49)	13.00 (10.01)	17.97 (9.69)
t, *p*		t^a^ = − 0.48, *p* = 0.62	t^a^ = − 0.90, *p* = 0.36	t^a^ = 0.13, *p* = 0.89	t^a^ = − 0.64, *p* = 0.53
Children and adolescent age group (years)					
0–5	92 (47.2)	47.50 (28.28)	15.43 (10.15)	13.97 (10.67)	18.08 (8.52)
06-Dec	63 (32.3)	37.44 (25.79)	10.19 (8.00)	11.77 (8.70)	15.60 (10.69)
13–18	40 (20.5)	45.50 (19.31)	12.50 (6.35)	13.60 (7.70)	19.40 (8.27)
F, *p*		F^b^ = 2.86, *p* = 0.05	F^b^ = 6.74, *p* = 0.001*	F^b^ = 1.04, *p* = 0.35	F^b^ = 2.31, *p* = 0.10
Age of the fathers	–	r^c^ = − 0.06, *p* = 0.42	r^c^ = − 0.11, *p* = 0.12	r^c^ = − 0.03, *p* = 0.64	r^c^ = − 0.02, *p* = 0.76
Age of the mother	–	r^c^ = − 0.13, *p* = 0.06	r^c^ = − 0.21, *p* = 0.003*	r^c^ = − 0.12, *p* = 0.07	r^c^ = − 0.04, *p* = 0.54
Age at cancer diagnosis	–	r^c^ = − 0.03, *p* = 0.71	r^c^ = − 0.07, *p* = 0.35	r^c^ = − 0.04, *p* = 0.57	r^c^ = − 0.03, *p* = 0.71
The number of hospitalizations of children and adolescents during the last 6 months	–	r^c^ = − 0.16, *p* = 0.03*	r^c^ = − 0.22, *p* = 0.005*	r^c^ = − 0.11, *p* = 0.15	r^c^ = − 0.14, *p* = 0.07
Number of previous chemotherapy	–	r^c^ = − 0.13, *p* = 0.16	r^c^ = − 0.11, *p* = 0.24	r^c^ = − 0.09, *p* = 0.35	r^c^ = − 0.17, *p* = 0.06

*Significant.

^a^Independent t-test.

^b^ANOVA.

^c^Pearson correlation coefficient.

As Table 1 shows, 55.2% of the children and adolescents with cancer were female. The mean number of previous chemotherapy was 10.87 (SD = 9.79). The number of hospitalizations of children and adolescents was 3.04 (SD = 3.76) with a range of 0–15 during the last six months. The child’s mean age at cancer diagnosis was 6.65 years (SD = 4.23).

### Mean scores of resilience, and hope, and their subscales as well as depression, anxiety, and stress

Table 2 shows the mean scores of Resilience Scale and Adult Hope Scale, and their subscales as well as DAS. The mean scores of Resilience Scale and Adult Hope Scale were 62.62 (SD = 16.93) and 50.61 (SD = 7.74), respectively. It showed that the Adult Hope Scale was in a moderate level. The mean and standard deviation of depression, anxiety, and stress were 13.00 (SD = 9.00), 13.0 (SD = 9.44), and 17.59 (SD = 9.16), respectively; the caregivers reported mild depression, moderate anxiety, and stress.

**Table 2:** The mean score of hope, resilience, and their subscales and depression, anxiety, and stress in the caregivers of children with cancer.

	Mean	Std. deviation
Adult hope scale	50.61	7.74
Hope subscales		
Agency	24.41	4.19
Pathway	26.23	4.49
Resilience scale	62.62	16.93
Resilience subscales		
Spiritual influences	6.19	1.97
Control	7.68	3.02
Positive acceptance of change and secure relationship	13.02	3.42
Trust in ones instincts tolerance of negative affect	15.44	5.23
Personal competence high standards and tenacity	20.15	6.37
Depression	13.00	9.00
Anxiety	13.08	9.44
Stress	17.59	9.16

### The association between caregivers’ DAS and the demographic and clinical characteristics of caregivers and their children and adolescents with cancer

Table 1 shows no significant difference between the mean score of DAS and caregiver’s gender, marital status, and education, and children and adolescent’s gender and age group (years). Moreover, no significant difference was found between DAS, the fathers, and mothers’ age, age at cancer diagnosis, and number of previous chemotherapies. On the other hand, the Pearson correlation coefficient showed a significant difference between the mean score of DAS and the number of hospitalizations of children and adolescents during the last 6 months (r = − 0.16, p-value = 0.03), (Table 1).

As shown in Table 1, a significant difference was observed between the mean score of depression and the caregiver’s marital status, age of mother, children and adolescents’ age group, and number of hospitalizations of children and adolescents during the least 6 months. Moreover, a significant difference was found between anxiety and father and mother’s education. In addition, a significant difference was observed between the mean score of caregivers’ stress and fathers’ education (Table 1). Therefore, based on the findings, some part of hypothesis 1 was approved.

### The association between DAS (depression, anxiety, and stress) and resilience, hope, caregivers’ gender, and the number of hospitalizations of children and adolescents

As Table 3 shows, a negative and significant association was observed between DAS, depression, anxiety and stress, and resilience and hope. Moreover, a positive and significant association was found between resilience and caregivers’ gender. Therefore, based on the findings, some part of hypothesis 2 was approved.

**Table 3:** The association between DAS (depression, anxiety, and stress) and resilience, hope, caregivers’ gender and the number of hospitalizations of children and adolescents.

		1	2	3	4	5	6	7	8
1. DAS		1							
2. Depression	r	0.93^b^	1						
	*p*	< 0.001							
3. Anxiety	r	0.93^b^	0.82^b^	1					
	*p*	< 0.001	< 0.001						
4. Stress	r	0.92^b^	0.79^b^	0.79^b^	1				
	*p*	< 0.001	< 0.001	< 0.001					
5. Resilience	r	− 0.48^b^	− 0.53^b^	− 0.43^b^	− 0.37^b^	1			
	*p*	< 0.001	< 0.001	< 0.001	< 0.001				
6. Hope	r	− 0.40^b^	− 0.41^b^	− 0.37^b^	− 0.33^b^	0.47^b^	1		
	*p*	< 0.001	< 0.001	< 0.001	< 0.001	< 0.001			
7. Caregivers’ gender	r	− 0.09	− 0.12	− 0.05	− 0.09	0.19^c^	0.08	1	
	*p*	0.21	0.11	0.48	0.22	0.01	0.25		
8. The number of hospitalizations of children and adolescents^a^	r	− 0.16^c^	− 0.22^b^	− 0.11	− 0.14	− 0.01	0.001	− 0.14	1
	*p*	0.03	0.005	0.15	0.07	0.81	0.99	0.09	

^a^During the last 6 months.

^b^Correlation is significant at the 0.01 level (2-tailed).

^c^Correlation is significant at the 0.05 level (2-tailed).

### Assess the multicollinearity

To assess the multicollinearity, we used variance inflation factor (VIF) values. The VIF above 10.00 shows that the predictors are multicollinear, and they would be strongly correlated. Based on the results of our study, the VIF of DAS with resilience, hope, gender, and the number of hospitalizations of children and adolescents during the last 6 months was 1.42, 1.39, 1.04, and 1.02, respectively.

### The moderator effect of caregivers’ gender in the relationship between resilience, DAS, and hope

As shown Figs. 1, 2, and 3 and SPSS macro PROCESS, model 1, no moderating effect of caregivers’ gender was observed in resilience and DAS (β = − 2.38, t-value = − 0.14, *p*-value = 0.88). Moreover, no moderating effect of caregivers’ gender was found in hope and DAS (β = 19.76, t-value = 0.76, *p*-value = 0.44). On the other hand, a moderating effect of caregivers’ gender was reported in hope and resilience (β = -44.57, t-value = -2.95, *p*-value = 0.003). It means that mother (effect = 0.59, t-value = 3.25, *p*-value = 0.001, CI = 0.23–0.94) and father (effect = 1.58, t-value = 6.90, *p*-value < 0.001) of children and adolescents with cancer significantly moderated the effect of hope on resilience. Therefore, based on the findings, hypothesis 3 was partially approved.

### The moderator effect of the number of hospitalizations of children and adolescents in the relationship between caregivers’ resilience, DAS, and hope

As shown in Fig. 4 and based on the SPSS macro PROCESS, model 1, no moderating effect of the number of hospitalizations of children and adolescents during the last 6 months was observed in resilience and DAS (β = − 0.66, t-value = − 0.32, *p*-value = 0.74). Moreover, no moderating effect of the number of hospitalizations was found in hope and DAS (β = 4.57, t-value = 1.49, *p*-value = 0.13). In addition, no moderating effect of the number of hospitalization was reported in the relationship between hope and resilience (β = − 0.67, t-value = − 0.34, *p*-value = 0.73). Therefore, based on the findings, hypothesis 4 was not approved. Thus, the number of hospitalizations of children and adolescents was not added in the next step and as a moderator.

### The mediator effect of hope in the relationship between resilience and DAS among caregivers of children and adolescents with cancer

According to Fig. 5 and based on the SPSS macro PROCESS, model 4, and Table 4, the direct effect of resilience significantly correlated with DAS (β = − 0.54, t-value = − 5.01, *p*-value < 0.001). DAS significantly correlated with hope (β = − 0.84, t-value = − 3.45, *p*-value = 0.0007). The indirect effect of resilience on DAS by hope was significant (Effect = − 0.18, SE = 0.06, 95% CI − 0.33 to − 0.06). Therefore, hypothesis 5 was approved.

**Table 4:** The mediate effect of hope in the relationship between resilience and DAS among caregivers of children and adolescents with cancer.

	Effect (β)	SE ^b^	t statistics	*p*-values	95% CI
Direct effect					
Resilience → DAS ^a^	− 0.54	0.10	− 5.01	< 0.001	− 0.75 to − 0.33
Hope → DAS ^a^	− 0.84	0.24	− 3.45	0.0007	− 1.32 to − 0.36
Resilience—> Hope	0.21	0.02	7.80	< 0.001	0.16–0.27
Indirect effects					
Resilience → Hope → DAS^a^	− 0.18	0.06 ^c^	–	–	− 0.33 to − 0.06

### The moderator effect of caregivers’ gender in the indirect effect of resilience on DAS through the mediating effect of hope

Based on Fig. 5 and the SPSS macro PROCESS, model 5, and Table 5, the direct effect of resilience significantly correlated with DAS (β = − 0.80, t-value = -2.39, *p*-value = 0.01). DAS significantly correlated with hope (β = -0.95, t-value = -3.33, *p*-value = 0.001). The indirect effect of resilience on DAS by hope was significant (Effect = − 0.20, SE = 0.08, 95% CI − 0.37 to − 0.04). This study showed DAS did not significantly correlate with gender among caregivers of children and adolescents with cancer (β = − 16.37, t-value = − 1.01, *p*-value = 0.31). No interaction was recognized between resilience and gender (R^2^ change = 0.004, F = 0.97, *p*-value = 0.32). However, based on Table 6 and the result of the moderation mediation model and conditional direct effects of resilience on DAS, female caregivers had a stronger correlation between resilience and DAS when compared to their male counterparts (β = − 0.56, t-value = − 3.90, *p*-value = 0.0001). Therefore, based on the findings, hypothesis 6 was approved.

**Table 5:** The moderated mediating effect of resilience on DAS in caregivers of children and adolescents with cancer.

	Effect (β)	SE ^b^	t statistics	*p*-values	95% CI
Direct effect					
Resilience → DAS^a^	− 0.80	0.33	− 2.39	0.01	− 1.46 to − 0.14
Hope → DAS^a^	− 0.95	0.28	− 3.33	0.001	− 1.51 to − 0.38
Resilience → Hope	0.21	0.03	7.08	< 0.001	0.15 to 0.28
Caregivers’ gender → DAS^a^	− 16.37	16.08	− 1.01	0.31	− 48.14 to 15.40
Indirect effects					
Resilience → Hope → DAS^a^	− 0.20	0.08 ^c^	–	–	− 0.37 to − 0.04

Hope as mediator, Caregivers’ gender as moderator.

^a^Depression, anxiety and stress.

^b^Standard Error.

^c^Bootstrapping Standard Error.

**Table 6:** Conditional effects of resilience on DAS by caregivers’ gender.

Caregivers’ gender	Effect	SE	t Statistics	*p*-values	95% CI
Mother	− 0.56	0.14	− 3.90	0.0001	− 0.85 to − 0.28
Father	− 0.33	0.20	− 1.58	0.11	− 0.74 to 0.08

## Discussion

This study demonstrated the mediating effect of hope and the moderating effect of caregivers’ gender on the relationship between resilience and DAS among caregivers of children and adolescents with cancer.

The result of our study showed that the mean score of resilience in caregivers of cancer patients was approximately similar to the studies conducted in Iran^26^ and China^25^. However, compared to a study in Turkey^33^, the mean score of caregivers’ resilience in our study was higher. Researchers reveal that lived experiences of resilience in parents of children with cancer mean “perceived competence, and social support, going through hardships and positive and negative experience of children’s disease”^34^.

In our study, caregivers reported mild depression, moderate anxiety, and moderate stress. Approximately, one-third of the parents with retinoblastoma patients reported depression and anxiety^35^. Another study revealed that roughly 10% of the parents of children with cancer had mild to severe depression disorder^9^.

This study indicated that the mean score of the caregivers’ hope was in a moderate level. Similarly, another study carried out on parents of retinoblastoma patients indicated that the score of hope was at a moderate level^35^. Caregivers of cancer patients were hopeful that the illness would be cured and that the survival span would be prolonged^16^.

In our study, the results of the direct analysis effect indicated that resilience negatively correlated with DAS. Similarly, studies reported that resilience was associated with depression and anxiety in caregivers of children and adolescents with cancer^19,20^. An integrative review showed resilience was associated with psychological distress and coping strategies in spousal caregivers of patients with cancer^12^. A higher level of resilience was associated with lower anxiety and depressive symptoms among family caregivers of patients with advanced cancer ^21^.

The results of the direct analysis effect on caregivers of children and adolescents with cancer showed that hope positively correlated with the caregivers’ DAS. A study indicated that hope had an important effect on anxiety and depression in family caregivers of advanced cancer patients^36^. In fact, hope, as a positive state, reduced depression in cancer patients^18^.

The results of the direct analysis effect of this study indicated that resilience positively correlated with hope in caregivers of children and adolescents with cancer. A study on cancer patients revealed that social support, hope, and resilience predicted the quality of life^37^.

Our study showed that hope mediated the relationship between resilience and DAS. Since no study had been conducted with the same findings as the present study, a comparison was made with other similar studies. Family support, hope, and resilience were associated with depressive symptoms in prostate cancer^38^. A systematic review has shown that the process of resilience begins with the diagnosis of cancer, and it might affect one’s mental well-being. Moreover, socio-cultural background and caregivers’ characteristics influenced this process^39^.

The result of the moderation mediation model indicated that female caregivers had a stronger correlation between resilience and DAS when compared to their male counterparts and hope mediated the relationship between resilience and DAS among caregivers of children and adolescents with cancer. This is a unique and innovative finding. Another study reported the association between symptom burden and anxiety and depression mediated by resilience in liver cancer patients^40^.

### Limitation

This study had some limitations. The type of children and adolescents with cancer that might affect the caregivers was not considered. Therefore, it is recommended that further studies should be conducted on a specific type of cancer. Moreover, this study was conducted on 200 caregivers with cancer; further studies with a larger sample size are suggested to improve the evidence-based practice.

### Implication for practice

One of the implications of this study is that the results could be effective for caregivers who take care of children and adolescents with cancer; conducting future research using interventions to increase the levels of hope and resilience is suggested. Since the caregivers’ anxiety and stress were at a moderate level, it is useful to provide supportive interventions, including the program to promote resilience and hope for caregivers who take care of children and adolescents with cancer. Another implication of this study is that it indicated that a portion of the relationship between resilience and DAS in caregivers of children and adolescents with cancer was mediated through hope, while resilience explained a portion of DAS that was independent of hope. This mediated moderator model indicated that the intermediate variable possibly confounded the relationship between resilience and DAS. Moreover, based on our findings, in follow-up studies one can predict DAS from the factors related to well-being, like resilience. Based on our findings, resilience, hope, and the moderator, which was caregivers’ gender, explained 24% of the variance in DAS; it means that many well-being factors could be used in these studies. Because the correlation between resilience and hope was high, one could choose any of them. Resilience is a good option, and hope could also be included.

## Conclusion

This study showed the direct effect of DAS and resilience with hope in the caregivers of children and adolescents with cancer. Moreover, the relationship between resilience and DAS was significantly mediated by hope in the caregivers of children and adolescents with cancer. In addition, female caregivers had a stronger correlation between resilience and DAS when compared to their male counterparts. It seems that resilient attitudes, female caregivers, and hope might provide a good protection against DAS.

## Data Availability

The datasets used and/or analyzed during the current study available from the corresponding author of this study on reasonable request.
